# Retrograde Intussusception after Roux-en-Y Gastric Bypass

**DOI:** 10.7759/cureus.8825

**Published:** 2020-06-25

**Authors:** Akshay Kumar, Stephanie Ogbonda, Purnadeo Persaud, Nimisha Shiwalkar

**Affiliations:** 1 Cardiothoracic Surgery, Lokmanya Tilak Municipal Medical College & General Hospital, Mumbai, IND; 2 Surgery, University of Texas Health Science Center at Houston, Houston, USA; 3 Surgery, Kansas City University of Medicine and Biosciences, Kansas City, USA; 4 Anesthesiology, Lokmanya Tilak Municipal Medical College & General Hospital, Mumbai, IND

**Keywords:** retrograde intussusception, gastric bypass surgery, laparoscopy

## Abstract

Retrograde intussusception (RI), although relatively uncommon, has been increasingly seen in adults post Roux-en-Y gastric bypass (RYGB) surgery. The exact mechanism for its occurrence remains unknown but several theories have attributed it to bowel persialtic dysmotility. The increase in bariatric surgery over the last decade has resulted in a proportionate increase in the number of cases of intussusception seen globally. We report a case of RI seven years following RYGB done for morbid obesity.

## Introduction

Retrograde intussusception (RI) is a rare long-term complication after Roux-en-Y gastric bypass (RYGB) (0.1%-0.3%), however, its etiology still remains unclear [1]. The origin of intussusception after gastric bypass is different from that of intussusception of other causes and is likely related to motility disorders in the divided small bowel, especially in the Roux limb [2]. This rare condition may cause obstruction and lead to bowel strangulation if not recognized and treated promptly. Contrast-enhanced computerized tomography (CECT) scan of the abdomen represents the diagnostic test of choice, however, a definite diagnosis cannot be established unless the patient undergoes surgical exploration [3]. For uncomplicated cases, where the bowel appears to be viable, simple reduction may suffice. Nevertheless, resection of the affected segment is recommended when bowel gangrene ensues.

## Case presentation

**Figure 1:**
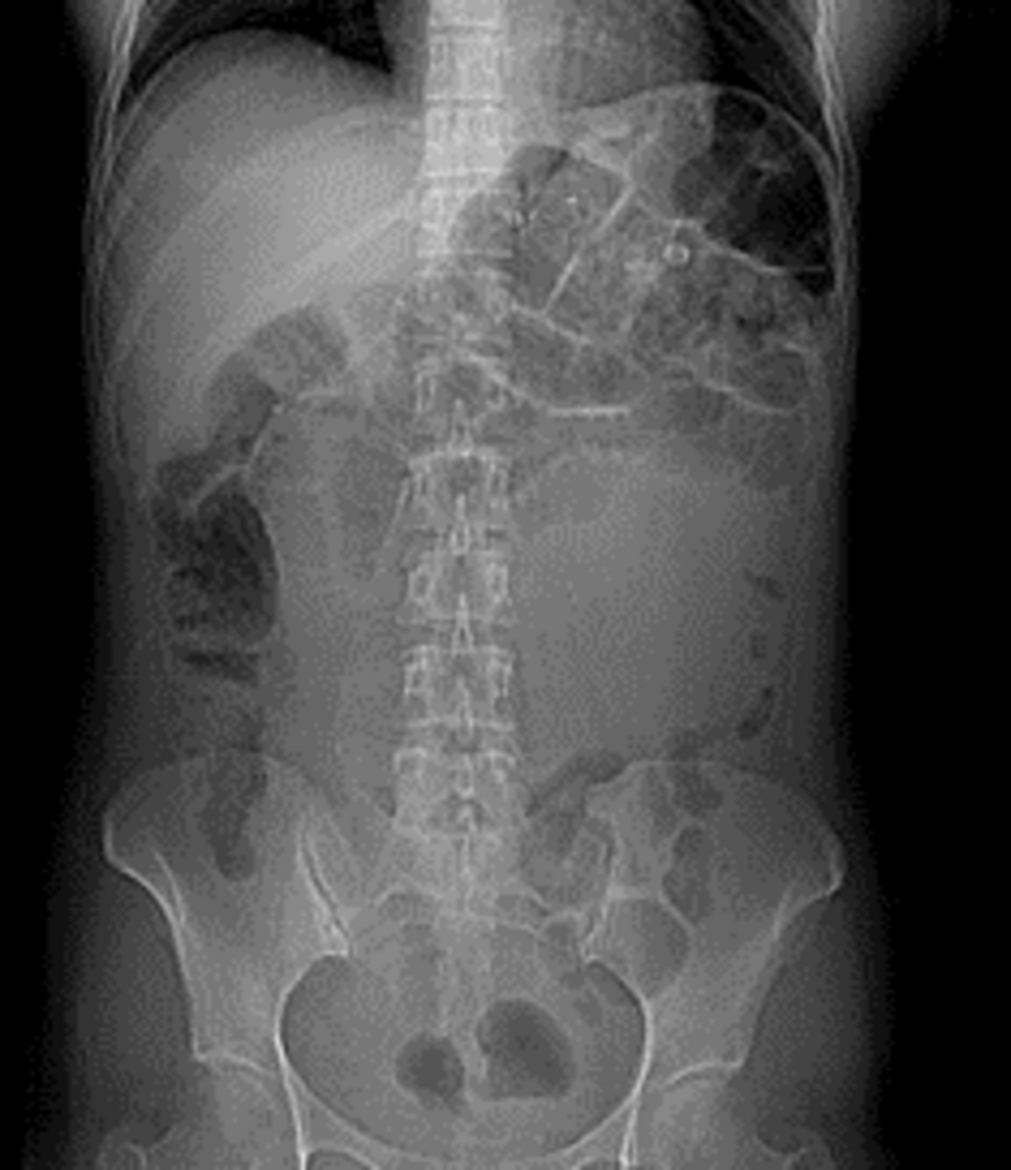
Upright X-ray abdomen showed dilated bowel loops in the left upper quadrant

A 47-year-old woman presented to the emergency department with epigastric abdominal pain, nausea, and non-bilious vomiting of one-day duration. The pain was predominantly in the upper abdomen and was associated with multiple bouts of non-bilious, non-bloody emesis. She was passing flatus and had bowel movement until one day prior to presentation. The patient reported no significant past medical history except for being operated for laparoscopic gastric bypass seven years ago. Her vitals were stable with a temperature of 36.7 C, heart rate of 67 beats per minute, blood pressure of 155/86 mmHg, and respiratory rate of 18/min. Physical examination revealed infraumbilical and epigastric scar from the previous laparoscopy. There was tenderness noted on palpation in the epigastric region without any guarding or rigidity. It was appreciated on the upright X-ray abdomen that there were multiple dilated small bowel loops in the left upper quadrant (Figure 1).

**Figure 2:**
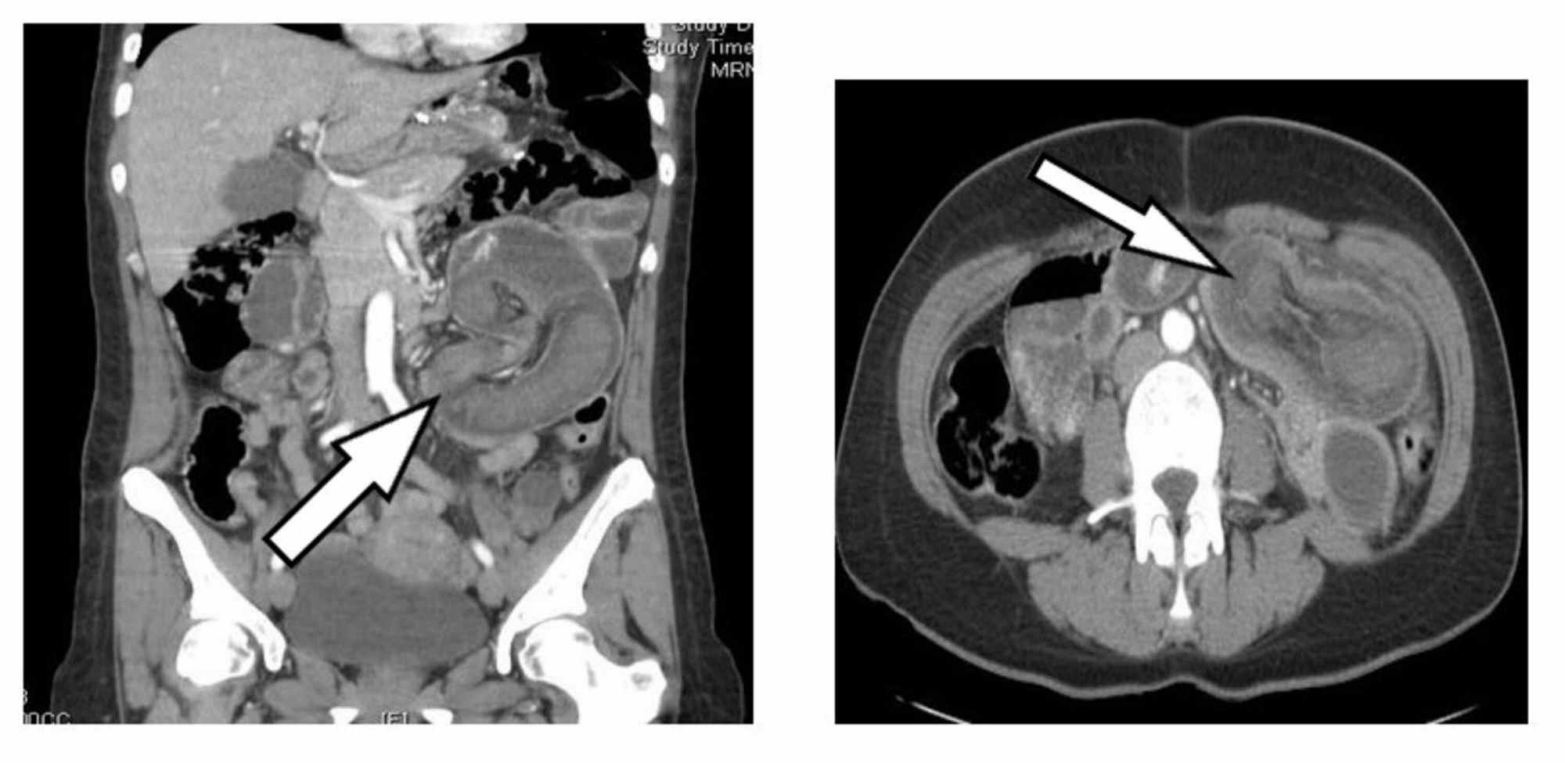
CECT scan of abdomen revealed bowel within bowel configuration in concentric rings (white arrow) in the left mid-abdomen suggestive of intussusception CECT: contrast-enhanced computerized tomography.

Her labs were within normal range except for white count of 11,400/mm3. CECT scan of abdomen revealed bowel within bowel configuration in concentric rings in the left mid-abdomen suggestive of intussusception (Figure 2).

**Figure 3:**
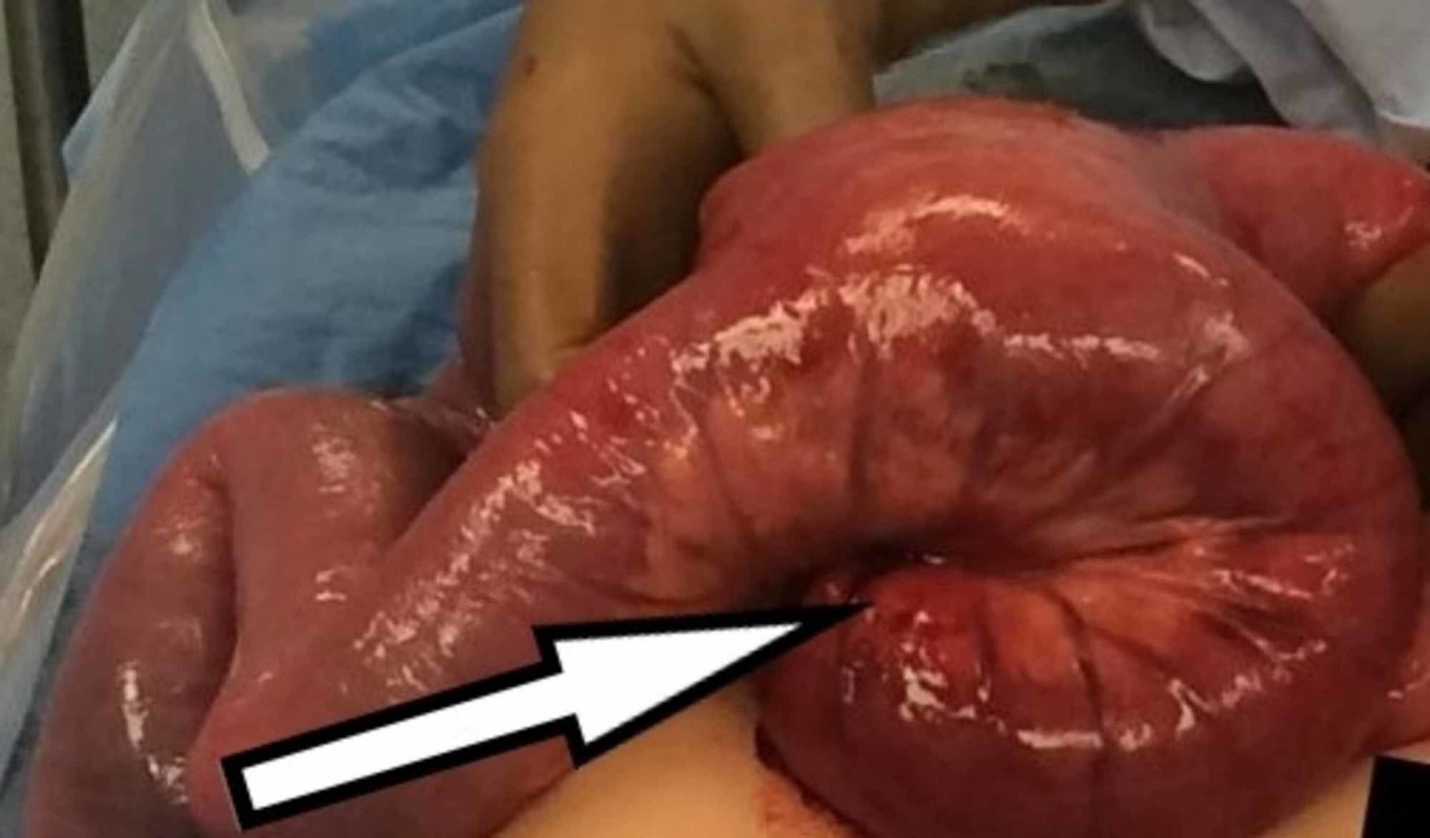
Intraoperative photo showing jejuno-jejunal intussusception (white arrow)

She underwent diagnostic laparoscopy which confirmed the diagnosis. Formal midline laparotomy with lysis of adhesions and reduction of intussusception was performed. However, the proximal small bowel was found to be non-viable and about 105 cm of the bowel from the jejunojenostomy was resected and continuity of bowel was achieved with side-to-side anastomosis using gastrointestinal anastomsis (GIA) stapling device (Figure 3). The common enterotomy was closed with 3-0 polydioxanone (PDS) in a running followed by 3-0 silk in an interrupted fashion. Postoperative recovery was uneventful, and she was discharged after tolerating full orals and return of bowel function.

## Discussion

Intussusception in adults is more frequently caused by structural lesions such as benign polyps, Meckel's diverticulum, intraluminal lipoms, and malignant neoplasms [4]. However, there is an increasing number of patients undergoing bariatric surgery procedures, retrograde or anti-peristaltic intussusception which has been identified as a long-term complication of RYGB surgery. RI in them typically occurs as telescoping of a distal bowel segment into the proximal segment at the site of jejuno-jejunostomy causing obstruction and if left untreated, in bowel strangulation or gangrene. Several case reports have described wherein RI occurs after both open and laparoscopic RYGB surgeries. The diagnosis of intussusception in this subsect of patients remains a significant challenge. The common symptoms presented by patients include abdominal pain, weight loss, constipation, nausea, and vomiting [5]. Physical examination could reveal tenderness in the epigastric or periumbilical region. Unlike in pediatric cases with intussusception, a palpable mass is not often discernible. The patient could also present with a scar indicating a previous bypass surgery and this should trigger the possibility of intussusception. The exact mechanism remains unknown; however, it is thought to be caused by peristaltic dysmotility which leads to small bowel obstruction. It is important to assess the viability of each bowel segment to prevent bowel ischemia and later stricture formation.

Animal studies replicating RYGB construction have shown that suppression of these ectopic pacemakers by either electrical pacing or by using an “uncut roux” prevents stasis by maintaining enteric myoneural continuity [6]. To date, the most widely accepted view has been that the creation of Roux limb disrupts the natural intestinal pacemakers in the duodenum and allows for the formation of ectopic pacemakers or migratory motor complexes in the Roux limb. It is believed that the electric potential generated by these ectopic pacemakers migrates in both the distal as well as the proximal limbs. This creates an area or segment of dysmotility, which according to some authors is responsible for developing intussusception in these patients [7]. 

The diagnostic tool most useful for detecting this condition is a CT scan. It reveals concentric rings which indicate a classic “target sign” identified in cases of intussusception. Surgical management is the most preferred treatment for intussusception which involves resection and anastomosis of the ischemic bowel. Despite treatment, the recurrence rate is about 0.2% with a mortality range of 1% to 16% [8].

## Conclusions

RI is an important cause of small bowel obstruction after bariatric surgery. Correct diagnosis and appropriate treatment in a timely manner can prevent mortality and morbidity.
